# The Effect of DNA-Dependent Protein Kinase on Adeno-Associated Virus Replication

**DOI:** 10.1371/journal.pone.0015073

**Published:** 2010-12-20

**Authors:** Young-Kook Choi, Kevin Nash, Barry J. Byrne, Nicholas Muzyczka, Sihong Song

**Affiliations:** 1 Department of Pharmaceutics, University of Florida, Gainesville, Florida, United States of America; 2 Department of Molecular Genetics and Microbiology, University of Florida, Gainesville, Florida, United States of America; 3 Department of Pediatrics, University of Florida, Gainesville, Florida, United States of America; New Mexico State University, United States of America

## Abstract

**Background:**

DNA-dependent protein kinase (DNA-PK) is a DNA repair enzyme and plays an important role in determining the molecular fate of the rAAV genome. However, the effect this cellular enzyme on rAAV DNA replication remains elusive.

**Methodology/Principal Findings:**

In the present study, we characterized the roles of DNA-PK on recombinant adeno-associated virus DNA replication. Inhibition of DNA-PK by a DNA-PK inhibitor or siRNA targeting DNA-PKcs significantly decreased replication of AAV in MO59K and 293 cells. Southern blot analysis showed that replicated rAAV DNA formed head-to-head or tail-to-tail junctions. The head-to-tail junction was low or undetectable suggesting AAV-ITR self-priming is the major mechanism for rAAV DNA replication. In an in vitro replication assay, anti-Ku80 antibody strongly inhibited rAAV replication, while anti-Ku70 antibody moderately decreased rAAV replication. Similarly, when Ku heterodimer (Ku70/80) was depleted, less replicated rAAV DNA were detected. Finally, we showed that AAV-ITRs directly interacted with Ku proteins.

**Conclusion/Significance:**

Collectively, our results showed that that DNA-PK enhances rAAV replication through the interaction of Ku proteins and AAV-ITRs.

## Introduction

DNA-PK is a nuclear serine/threonine protein kinase that consists of a 460 kDa catalytic subunit (DNA-PKcs) and a heterodimer (Ku70 and Ku80). DNA-PK plays important roles in DNA repair and V(D)J recombination through nonhomologous end joining (NHEJ). When DNA-PK encounters DNA lesions such as DNA double strand break (DSB) damage by ionizing radiation, Ku70/80 binds with high affinity to DNA ends independent of their end sequence or structure [1], [2], [3]. The Ku heterodimer recruits DNA-PKcs to form an active DNA-PK holoenzyme. LigaseIV/XRCC4 interacts with DNA-PK on DNA ends, which leads to NHEJ [4], [5]. Several proteins including Mre11/Rad50/Nbs1 and Artemis are involved in this process [6], [7]. Activity of DNA-PKcs may be regulated by autophosphorylation of DNA-PKcs at seven putative phosphorylation sites including Thr^2609^ and Ser^2056^ [8], [9]. Cells or animals lacking DNA-PK functions are deficient in a protective response to ionizing radiation and various radiomimetic agents [10], [11]. DNA-PK is a potential target protein in many cancer therapeutics since inhibitors of DNA-PK can selectively sensitize tumor cells to ionizing radiation. Wortmannin, an inhibitor of PI 3-kinase, inhibits DNA-dependent protein kinase and sensitizes cells to ionizing radiation (IR) [12], [13]. In addition, wortmannin directly binds to the kinase domain of DNA-PKcs and inhibits the function of DNA-PKcs noncompetitively [14]. DNA-PK is a sensor molecule that determines the cellular fates by regulating cellular proteins related with cell cycles, DNA repair, and apoptosis [9], [15], [16], [17]. Paradoxically, the Ku70/80 complex can also inhibit nonhomologous end joining when it binds to the telomere complex, shelterin [18].

Adeno-associated virus (AAV) is a nonpathogenic human parvovirus that contains a linear single-stranded DNA (ssDNA) genome [19]. The AAV genome encodes two large open reading frames, *rep* and *cap*, that are flanked by 145 nucleotide inverted terminal repeats (ITRs). AAV has an interesting biphasic life cycle, either productive infection in the presence of a helper virus, e.g., adenovirus or herpes simplex virus (HSV), or latent infection in the absence of a helper virus. The ITRs comprise the Rep binding elements (RBE and RBE') and the terminal resolution site (*trs*) and form a T-shaped hairpin structure that serves as the primer for minimal origin of AAV DNA replication and for the site specific nicking of the AAV ITR at the *trs* that is required for repairing covalently closed ITRs during AAV replication [20], [21], [22], [23]. The large Rep proteins (Rep68 or Rep78) mediate viral DNA replication and *trs* nicking [20], [24], [25], [26], [27] and regulate AAV gene expression [28], [29], [30], [31], [32], [33], [34] and packaging [35], [36]. Rep68 or Rep78 also play important roles for site-specific integration of wild type AAV2 into human chromosome 19q13.3qter, named the AAVS1 locus [37], [38], [39]. AAV DNA replication requires the ITR, cellular polymerases, and helper virus-derived factors. The p5 promoter region that regulates rep gene expression is also involved in a reduced Rep-dependent replication and site-specific integration that occurs in the absence of the ITR and relies on the RBE and cryptic *trs* in the p5 promoter [40].

In addition to the Rep proteins and ITRs, AAV DNA replication requires cellular proteins and helper virus-derived factors depending on the helper virus used. In the presence of Ad, *in vitro* replication assays suggest that four cellular complexes are essential for AAV DNA replication; these are polymerase δ, proliferating cell nucelar antigen (PCNA), replication factor C (RFC), and minichromosome maintenance complex (MCM) [25], [41], [42], [43]. The Ad and cellular single stranded DNA binding proteins (DBP and RPA) have also been shown to stimulate AAV DNA replication *in vitro* [43], [44] [41]. Rep has been shown to interact with all of these cellular and viral proteins [44], [45].

In contrast to Ad helper infections, relatively little is known about cellular proteins involved in AAV replication during coinfection with Herpes simplex virus (HSV). The herpes helicase primase complex and single stranded DNA binding protein are essential for promoting AAV DNA replication *in vivo* at a minimal level [44], [46], [47]. However, expression of the HSV DBP and helicase/primase provide only 10% of the normal DNA replication seen with wild type herpes coinfection [47]. This suggests that other herpes genes provide essential functions and some of these have recently been identified, (ICP0, ICP4, ICP22) [48]. The herpes DNA polymerase, which appears to provide partial helper function under some conditions [46], [48], has also been shown to be completely dispensable for rAAV production [49], suggesting that a cellular polymerase may be necessary in the presence of Herpes coinfection.

We have previously reported that DNA-PK is involved in determining the molecular fate of the rAAV genome in skeletal muscle and in liver [50], [51]. However, the effect of DNA-PK on AAV replication remains elusive. Nash et al (44) have recently shown that Ku70/80 complex, which forms a complex with DNA-PK, as well as DNA-PK, both form a complex with Rep78 during productive infections in the presence of adenovirus. They also showed that the DNA-PK/Ku70/Ku80 complex stimulates AAV DNA replication *in vitro*, although it does not appear to be essential. In this report we examine the effect of DNA-PK/Ku70/80 on AAV DNA replication in the presence of HSV and Ad. In order to test the effect of DNA-PK on rAAV replication, we have employed both *in vivo* and *in vitro* replication assays. We demonstrated that inhibition of DNA-PK by wortmannin or siRNA resulted in decrease of rAAV replication in MO59K and 293 cells in the presence of Herpes virus and in 293 cells in the presence of Ad helper functions. We also confirmed that depletion of Ku proteins lead to a reduction of AAV replication in an *in vitro* assay using Ad infected extracts.

## Results

### DNA-PK inhibitor, wortmannin Inhibits rAAV replication

**Figure 1:**
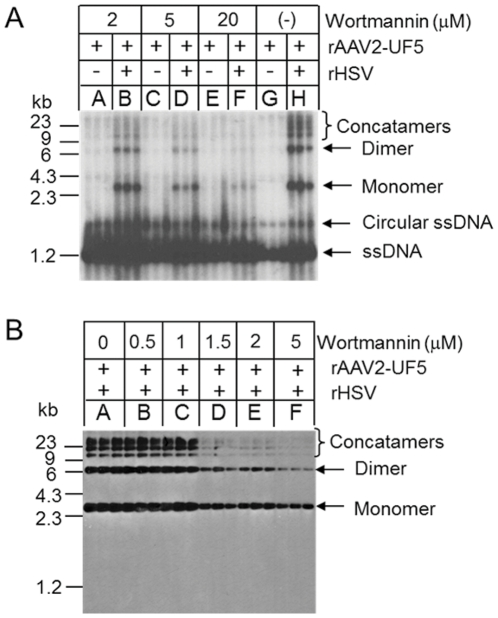
rAAV replication in MO59K cells and 293 cell treated with wortmannin. Hirt DNA was purified two days after viral infection and subjected to Southern blot analysis. All samples were triplicated and hybridized with ^32^P labeled CMV probe. Replicated forms of rAAV include double-stranded monomer (about 3.4 kb), or dimer (about 6.8 kb), and concatamers DNA (high molecular weight). Notice that treatment of wortmannin reduced rAAV replication in a dose-dependent manner in both MO59K cells (A) and in 293 cells (B).

In order to test the effect of DNA-PK on rAAV replication, we first employed wortmannin, an inhibitor of DNA-PK [14]. DNA-PK positive (+/+) cell lines, MO59K and 293 cells were infected with rAAV2-UF5 with or without recombinant herpes helper virus (containing the AAV2 *rep* and *cap* genes) [52]. Cells were treated with different concentrations of wortmannin and two days after viral co-infection, episomal DNA (Hirt DNA) was isolated. Replicated forms of rAAV-UF5 DNA including double-stranded monomer (about 3.4 kb), double-stranded dimer (about 6.8 kb) and high molecular weight concatamers (over 13 kb) were analyzed by Southern blot analysis using ^32^P labeled vector specific (CMV) probe. As shown in Figure 1, treatment of wortmannin decreased the rAAV genome replication in dose-dependent manner in both cell lines. No significant cytotoxicity was observed when wortmannin concentration was below 20 µM in MO59K cells, or below 5 µM in 293 cells. We noticed that the conversion of single-stranded AAV genome to double-stranded monomer or concatamers was more efficient in 293 cells than in MO59K cells, probably due to E1 gene expression in 293 cells [53], [54], [55].

### Targeting DNA-PKcs mRNA inhibits rAAV replication

**Figure 2:**
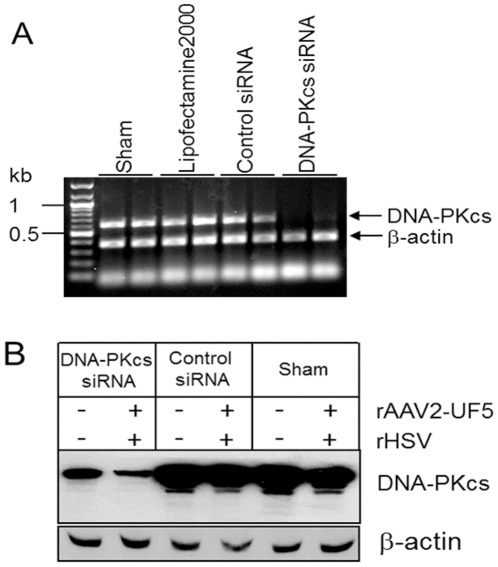
Targeting DNA-PKcs by siRNA. 293 cells are transfected with siRNA (100 pmole) targeting DNA-PKcs mRNA and non-specifically control siRNA using Lipofectamine™ 2000. Two days after transfection, cells were harvested. (A) RT-PCR for the detection of DNA-PKcs mRNA. Amplified cDNA fragments were separated on a 1% agarose gel. (B) Western blot for the detection of DNA-PKcs protein using beta-actin protein as an internal control.

**Figure 3:**
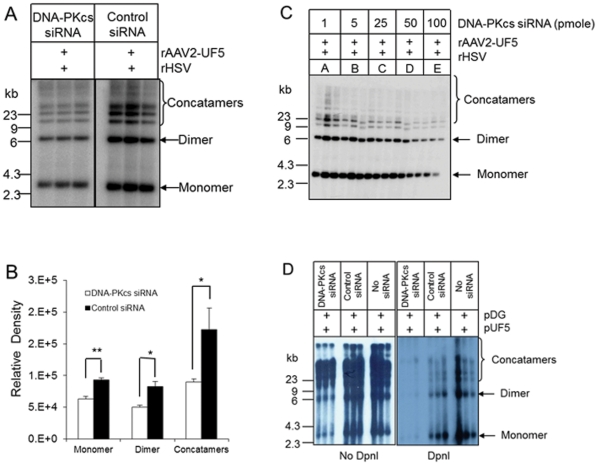
Targeting DNA-PKcs reduced rAAV replication. 293 cells were transfected with DNA-PKcs specific siRNA (DNA-PKcs siRNA) or control siRNA. Two days after transfection, cells were infected with rAAV-UF5 and rHSV, or transfected with pUF5 and pDG. rAAV replication was evaluated by Southern blot analysis as described in Figure 1. (A) Effect of DNA-PKcs siRNA on rAAV replication by Southern blot after vector infection. Concentration of DNA-PKcs siRNA and control siRNA was 100 pmole. (B) Densitometry analysis of data from Figure A. **, *P<0.001* for monomer; *, *P<0.05* for dimer and concatamers when compared with control siRNA group. (C) Dose dependent effect of DNA-PKcs siRNA on rAAV DNA replication. (D) Effect of DNA-PKcs siRNA on rAAV after vector and helper plasmid transfection. Left panel, without DpnI digestion; Right panel after. Note: DpnI digestion removes transfected plasmid DNA and shows all *de novo* replicated rAAV forms (monomer, dimer and concatamers).

The results above indicated that DNA-PK may play a role in enhancing rAAV replication. However, wortmannin inhibits not only DNA-PK, but also other protein kinases, such as phosphatidylinositol 3-kinase (PI-3). In order to pinpoint the role of DNA-PK, we employed synthetic siRNA to target DNA-PKcs mRNA. Synthetic siRNA at 100 pmole significantly decreased mRNA (Figure 2A) and protein levels (Figure 2B) in 293 cells, while control siRNA or Lipofectamine™ 2000 reagent alone showed no effect. These results demonstrated that siRNA was efficient for inhibition of DNA-PKcs. As shown in Figure 3A and B, inhibition of DNA-PKcs by siRNA resulted in a significant decrease of rAAV replication as compared with control siRNA treatment. The inhibitory effect of the siRNA on rAAV replication was dose dependent (Figure 3C). Since the rAAV genome is linear and single stranded DNA, the viral infection may affect DNA-PK activity [56], [57], [58]. To avoid the unwanted effect from viral transduction of rAAV and rHSV [59], we evaluated rAAV replication using transfected double stranded vector and helper plasmids. Twenty-four hours after siRNA transfection, 293 cells were co-transfected with pDG, which supplies all of the Ad helper genes, and pUF5. Two days after the transfection, Hirt DNA was isolated and digested with *Dpn*I to remove plasmid DNA. Results from this study again showed that inhibition of DNA-PK decreased rAAV replication (Figure 3D).

### Molecular analysis of replicated rAAV DNA

**Figure 4:**
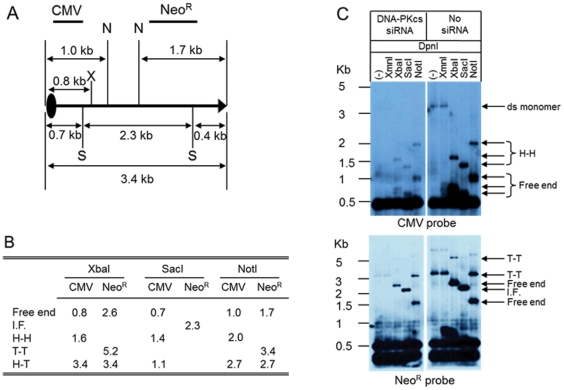
Structure analysis of replicated DNA. (A) Map of the rAAV-UF5 vector; X (XbaI), S (SacI), and N (NotI), Two bold lines indicate the position of CMV and Neo^R^ probes. (B) All possible genome sizes generated from different AAV junctions. I. F., internal fragment; H-H, head-to-head; T-T, tail-to-tail; H-T, head-to-tail. (C and D) Southern hybridization probed with CMV (C), and Neo^R^ (D) after restriction enzyme digestion of Hirt DNA.

We and others have previously shown that in latent infections, rAAV DNA forms mainly head-to-tail (H-T) junctions and persists as an episome in liver and muscle cells. Cellular enzymes, such as DNA-PK play important roles in the junction formation and persistence of AAV DNA [50], [51] and would be expected to produce circular rAAV genomes with head to tail junctions. Although AAV is known to replicate by a hairpin priming mechanism, a rolling circle mechanism has been proposed during the establishment of latent infections [60], [61]. Unlike the self-priming model, which generates head-to-head (H-H) or tail-to-tail (T-T) junctions, the rolling circle model generates head-to-tail (H-T) junctions. In order to evaluate the structures of replicated rAAV junction and the effect of DNA-PK on junction formation, we digested Hirt DNA with unique restriction enzymes, *Xba*I (1-cutter), *Sac*I (2-cutter), and *Not*I (2-cutter) as shown in Figure 4A. All of the possible fragments generated from these digests are listed in Figure 4B. Southern blot analysis using two different probes showed that all predicted fragments (H-H, T-T, free ends) were detected, except H-T junctions (Figure 4C). The low or undetectable levels of head to tail junctions suggested AAV-ITR self-priming was still the primary mechanism for AAV DNA replication. Inhibition of DNA-PK did not affect the type of AAV junction formed during AAV replication.

### In vitro AAV replication

**Figure 5:**
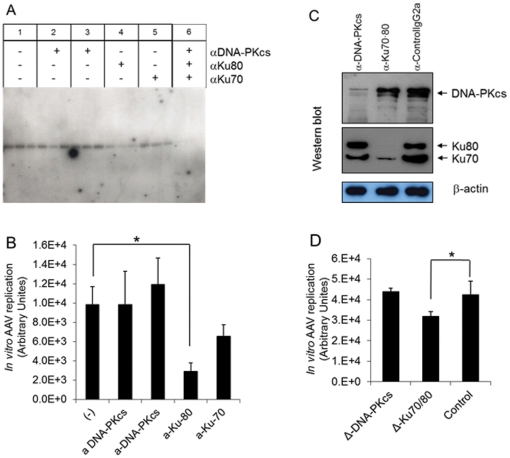
In vitro AAV replication. (A) *In vitro* AAV replication using nuclear extract (NE) supplemented with anti-DNA-PKcs Ab-2_3198–4127_ (2, *), anti-DNA-PKcs Ab-4_cocktail_ (3, **), anti-Ku80 (4), anti-Ku70 (5), or with all antibodies (6). (B) Densitometry analysis of result from Figure 5A. Supplement of anti-ku80 decreased AAV replication by 30% (DpnI-resistant AAV DNA, *P<0.05* compared to sham control). (C) Western analysis shows the depletion of DNA-PKcs, and Ku70/80. (D) In vitro AAV replication using DNA-PKcs (Δ-DNA-PKcs) or Ku70/80 (Δ-Ku80/70) depleted NE. Relative density of each treatment (n = 3) is plotted. * P<0.05.

In order to confirm the observation that inhibition of DNA-PK decreased rAAV replication in cells, we employed a previously developed in vitro replication assay to explore the role of DNA-PK and Ku70/80 [25], [43]. In this assay, the reaction contains purified Rep 68, dsAAV DNA template with covalently closed ends and nuclear extract (NE) from adenovirus-infected HeLa cells. Since HeLa cells express high levels of DNA-PK, we tested the effect of selective inhibition of each subunit of the DNA-PK complex by adding anti-DNA-PKcs, anti-Ku70, or anti-Ku80 antibodies to the HeLa NE before starting the AAV replication reaction. As shown in Figure 5A, addition of anti-Ku80 antibody significantly inhibited rAAV replication (70%, *P<0.05*), while anti-Ku70 antibody showed a moderate decrease of rAAV replication (33%, *P = 0.074*). However, we did not observe inhibition of AAV replication using anti-DNA-PKcs antibodies (Ab-2_3198–4127_ or Ab-4_cocktail_). In order to rule out a non-specific effect of the antibodies on AAV replication, we performed the assay with HeLa nuclear extracts that had been depleted for DNA-PKcs or Ku heterodimer. Specific depletion of DNA-PKcs by anti-DNA-PKcs Ab-4_Cocktail_ and Ku proteins by anti-Ku70/80 (Ku-Ab3) was observed by Western blotting (Figure 5C). As shown in Figure 5D and 5E, depletion of Ku70/80 decreased AAV replication (*P<0.05* by one-tailed distribution), while depletion of DNA-PK-cs did not. The results from these *in vitro* replication studies confirmed the previous report that Ku70/80 interacts with AAV Rep protein in vivo, and that they can enhance in vitro AAV DNA replication and partially substitute for MCM complex [45].

### AAV-ITRs interact with Ku proteins

**Figure 6:**
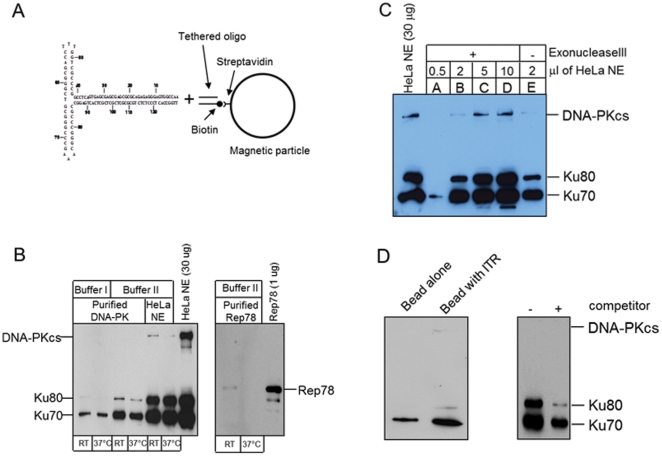
AAT-ITR interacts with Ku proteins. (A) construction of AAV-ITR on a magnetic particle. (B) ITR on magnetic bead interacts with Rep78 and Ku proteins. AAV-ITR was bound to purified proteins or HeLa nuclear extract (NE) at room temperature or 37°C and then subjected to western blot analysis using antibodies toDNA-PKcs, Ku80 and Ku70. Rep78 are used as a positive control. (C) T-shaped closed ITR interact with Ku proteins in dose-dependent manner. AAV-ITRs on the bead were treated with Exonuclease III and incubated with different amount of HeLa NE (65µg/µl). The ITR binding proteins were subjected to western blot analysis for DNA-PKcs, Ku80 and Ku70. (D) Competition assay (right panel). When AAV-ITR on bead was incubated with HeLa nuclear extract, free AAV-ITR was added (2.5 fold) as a competitor. Streptavidin-coated magnetic beads and the beads with AAT-ITR (left panel) served as a control and showed addition of AAV-ITR increased pull down of Ku proteins.

In order to test for a direct interaction between the AAV ITR and DNA-PK, we built an AAV-ITR by annealing and ligating three synthetic oligonuceotides. This ITR was then linked to a magnetic particle (Figure 6A), and as expected, the ITR interacted with Rep 78 (Figure 6B, right panel). In addition, using the ITR coated magnetic particles, we successfully pulled down and isolated Ku70 and Ku80 proteins (Figure 6B, left panel). To eliminate the possibility that incomplete ITRs or single stranded DNA interacted with the Ku proteins, we treated the ITR coated beads with exonuclease III to remove any partially assembled ITRs. As shown in Figure 6C, exonuclease III treatment did not affect the interaction between the AAV-ITR and Ku proteins. The interaction was also found to be a function of the concentration of Ku proteins. Finally, when free competitor AAV-ITR was added to the reaction, less Ku protein was pulled down (Figure 6D right) although the streptavidin-coated beads alone weekly interact with Ku 70 (Figure 6D left). These results demonstrated that Ku proteins can directly bind to hairpined AAV-ITRs, even in the absence of Rep protein.

## Discussion

AAV replication has been intensively investigated and the contributions of AAV ITR sequences and Rep proteins have been reasonably well defined [20], [21], [22], [23], [25], [62], [63], [64], [65], [66], [67], [68]. However, the effects of cellular proteins on AAV replication are not completely understood. Although the minimum set of proteins required to replicate AAV DNA efficiently *in vitro* has been identified for both Herpes and Ad infected cells [41], [42], [43], [44], [46], [47], [49], [69], [70], [71], it has recently been reported that Rep protein interacts with 188 cellular proteins [45]. Some of these Rep-interacting cellular factors play important roles in cellular DNA replication or repair and are likely to have a role in AAV DNA replication as well. Understanding these mechanism(s) will enable us to enhance rAAV vector production and to develop a safe gene delivery system. In the present study, we focused on the role of the DNA-PK complex (DNA-PKcs and Ku70/80) in AAV DNA replication.

Our results showed that reduction of DNA-PK at the protein or RNA levels decreased rAAV replication, suggesting that one or more components of the DNA-PK complex can enhance AAV replication. *In vivo*, long-term inhibition of DNA-PKcs by wortmannin and siRNA reduced rAAV replication in both MO59K and 293 cells (Figs. 1, 2 and 3), indicating that DNA-PKcs plays an important role in rAAV replication. Furthermore, DNA-PKcs siRNA reduced AAV DNA replication when either Herpes virus or Ad helper functions were used (Fig. 3). However, when cell-free extracts were used in an *in vitro* replication assay, depletion of DNA-PKcs did not affect rAAV replication suggesting that the effect of DNA-PKcs observed *in vivo* was indirectly through acting on downstream factors, such as Ku proteins. Indeed, our results showed that Ku70 and Ku80, had a clear effect in vitro when they were depleted by either antibody precipitation or antibody addition (Fig. 5). Finally, we showed that the components of the DNA-PK complex, particularly Ku70/80, could bind to a hairpinned AAV ITR. Because the magnetically tagged substrate used in these binding studies had a DNA end that was blocked with a magnetic bead, our results showed the direct interaction between Ku proteins and close-ended AAT-ITR. These results are consistent with observations reported previously using a ChIP assay [72]. Our results also are consistent with the recent report by Nash et al [45], who showed that purified Ku70/80 could partially substitute for the MCM helicase complex in an AAV DNA replication assay. This group also showed by antibody co-precipitation that Ku70/80 formed a complex with Rep78 and 68 *in vivo* that was independent of the presence of DNA. Together, these results suggest that the enhancing effect of DNA-PKcs that we observed *in vivo* was probably through phosphorylation of Ku proteins, and suggested that Ku protein activated by DNA-Pkcs stimulated AAV replication *in vitro* in the absence of DNA-PKcs.

DNA-PK is a DNA repair enzyme consisting of a large catalytic subunit (DNA-PKcs), which has ser/thr kinase, and a heterodimeric complex consisting of Ku70 and Ku80 [73]. The Ku heterodimer associates tightly with double stranded DNA breaks and recruits DNA-PKcs, XRCC4 and Ligase IV to repair the DNA break by non-homologous end joining [74], [75], [76]. Ku also binds to telomeres via a high affinity interaction with TRF1, a component of the telomere shelterin complex and has a role in maintaining the stability of telomeres [18]. In contrast to its role in DNA repair, when Ku binds telomeres, it prevents telomere end joining. Finally, Ku has also been shown to have a 3′ to 5′ helicase activity and ATPase activity [77] and these activities appear to be unrelated to its binding of DNA ends.

We and others have shown that DNA-PK plays an important role in processing recombinant AAV genomes [51], [72], [78], [79], [80], [81]. Injection of rAAV into scid mice (DNA-PKcs negative) showed a persistence of linear episomal rAAV DNA containing free ends in mouse tissue, in contrast to normal mice, in which all of the free ends had been joined predominantly in a head to tail fashion [78], [79]. This result was consistent with the primary role of the DNA-PK complex in NHEJ, and suggested that this mechanism promoted rAAV DNA circulization, a key step in establishing stable transduction. Curiously, Zentilin et al [72] showed that in short term cell culture experiments (48 hrs), transduction was increased in Ku80 negative cell lines in the presence of hydroxyurea, suggesting that alternative mechanisms for forming transducing genomes can exist in the absence of DNA synthesis. In contrast, the results reported here showed that AAV DNA synthesis in the presence of helper virus functions did not result in circular intermediates or head to tail junctions, regardless of whether Ku70/86 was activated by DNA-PKcs (Fig 5). This suggests that the primary role of Ku70/80 in stimulating AAV DNA replication is unrelated to its role in promoting NHEJ or transduction. Our results are consistent with the recent report that AAV replication in the presence of Ad coinfection stimulated a DNA damage response that was primarily due to DNA-PKcs [58].

Given its inhibition of telomere end joining and its DNA helicase activity, we can suggest two general mechanisms by which Ku70/80 might stimulate AAV DNA synthesis. First, the interaction with Rep and the ITR may prevent NHEJ of AAV DNA replicative intermediates. This would be consistent with the finding that the protein complex containing the Ad E4 orf 6 and E1b 55K proteins targets the Mre11/Rad50/Nbs1 complex (MRN) for degradation [82]. In the absence of these two Ad helper functions for AAV (E1b55K and E4orf6), Ad DNA forms concatemers, thereby inhibiting Ad DNA replication. The MRN complex also localizes to AAV replication centers in the presence of Ad coinfection and inhibits AAV DNA replication in the absence of the E4orf6/E1b complex [83]. Therefore, the helper function provided by E1b and E4orf6 is believed in part to be the inhibition of MRN mediated NHEJ during AAV DNA replication. By binding to AAV ITRs, Ku70/80 may also prevent interaction of the ITR with MRN. Furthermore, by binding to the Rep-ITR complex, Ku itself may be prevented from recruiting the other components of the DNA-PK NHEJ pathway. Thus, binding of Ku to the ITR and Rep would inhibit the two major NHEJ pathways in mammalian cells.

Ku70/80 may also play a role in strand displacement synthesis. We have shown previously that AAV DNA replication can be reconstituted *in vitro* with purified proteins, including Rep, pol δ, PCNA and RFC [41], [42]. Here and elsewhere, we have also shown that replication can be at least partially reconstituted by substituting Ku for MCM [45]. Both proteins have similar 3′ DNA helicase activities that prefer a replication fork as a substrate [77], [84]; thus, both could function in AAV DNA replication as strand displacement helicases. Neither has activity on blunt ended DNA molecules [77], [84] and, therefore, the initial melting of AAV ends may be the function of Rep, which can bind to a Rep binding element within the ITR and unwind the end [85]. Once a nascent fork is established, either Ku or MCM can load onto the 3′ strand and unwind the rest of the ITR to form the hairpin primer required for loading DNA polymerase and executing strand displacement synthesis.

In summary, we have presented evidence both *in vivo* and *in vitro* that the DNA-PK complex and in particular Ku70/80 stimulates AAV DNA replication in the presence of both Ad and Herpes coinfection. We have also suggested two possible mechanisms that might account for this activity and guide future experiments.

## Materials and Methods

### Cells and Reagents

A human glioma cell line (MO59K) was obtained from ATCC and was cultured in DMEM/F-12 medium (Cellgrow).Human embryonic kidney 293 cells (Microbix) were cultured in Dulbecco's Minimal Essential Medium (DMEM). All media were supplemented with 10% fetal bovine serum (Cellgro), penicillin (100 U/ml) and streptomycin (100 µg/ml). Wortmannin (Sigma) was dissolved in DMSO at 10 mM and stored at −80°C.

### rAAV transduction

The rAAV2-UF5 vector was produced at the University of Florida Gene Therapy Center as described previously [86]. This vector contains green fluorescent protein (GFP) cDNA driven by cytomegalovirus (CMV) promoter. The titer of the rAAV-UF5 used in this study was 2.5×10^13^ physical particles/ml (1×10^12^ infectious unit/ml). To test AAV replication from viral DNA, cells were infected with rAAV2-UF5 at 1000 particles/cell or co-infected without recombinant HSV helper vector which contains AAV *rep* and *cap* genes [52]. To test AAV replication from plasmid DNA, cells were transfected 1.2 µg of helper plasmid (pDG), and 0.8 µg of pTR-UF5 by using Lipofectamine 2000™ (Invitrogen). Hirt DNA was purified two days after viral infection and was used for Southern blot analysis to detect replicated forms of AAV genome. The densitometic quantification of AAV genomes was performed with Kodak Gel Image Software.

### siRNA transfection

Two double stranded RNA molecules were purchased from Ambion (Austin, TX). The siRNA for DNA-PKcs (5′-GAU CGC ACC UUA CUC UGU U-3′) targets the sequences of 352 base and downstream sequences of human DNA-PKcs mRNA [87]. Silencer™ Negative Control #2 siRNA from Ambion which does not induce nonspecific effects on gene expression was used as a transfection control. 293 cells at the number of 2.5×10^5^ cells/well were cultured in 6-well plates and transfected with 100 pmole of siRNA using Lipofectamine™ 2000 (Invitrogen). Next day, siRNA transfected cells were infected or transfected to test AAV replication as described above.

### RT-PCR

Total RNA was extracted from siRNA treated cells using Trizol™ reagent (Invitrogen) and RT-PCR was performed with Access™ RT-PCR kit (Promega) using 1 µg total RNA. Primers were derived from the coding region of DNA-PK cDNA (upstream primer: 5′-ACT GAC ACA GAC TGC AGA TGG AAG-3′, downstream primer: 5′-AGG GTG GAA AGA AAG AGA AGG TGG-3′). Beta-actin was used as a control (upstream primer: 5′-TCA CCA TGG ATG ATG ATA TCG CCG-3′, downstream primer: 5′-ACA TGA TCT GGG TCA TCT TCT CGC). Synthesis of the first strand cDNA was performed at 48°C for 45 min. The PCR were performed at 96°C for 1 min, 56°C for 1 min and 72°C for 2 min for 35 cycles. The amplified PCR products were fractionated on a 1% agarose gel.

### Western blot analysis

For western blot analysis, cell pellets were lysed in M-PER™ mammalian protein extraction reagent (Pierce) supplemented with protease inhibitors (leupeptin, pepstatin, aprotinin, antipain, 1 µg/ml each) and PMSF (at 1 mM). The protein concentration was determined using BCA kit (Pierce). Thirty micrograms of total cellular proteins were separated on 8% SDS-polyacrylamide gel and transferred onto a nitrocellulose membrane (Amersham). The mouse anti-DNA-PKcs (DNA- PKcs Ab-4_1–4127_) or mouse anti-Ku80 (Ku Ab-2_610–705_) or mouse anti-Ku70 (Ku Ab-4_506–541_) (all antibodies were purchased from NeoMarkers) was used to detect DNA-PKcs, or Ku80 or Ku70 respectively. Horseradish peroxidase-conjugated goat anti-mouse IgG was then used and the signal was detected using ECL (Amersham).

### In vitro AAV replication assay

In order to inhibit DNA-PK subunits, 25 µg (1 µl) of Ad-infected HeLa cell nuclear extract was preincubated with 1 µl of specific antibodies against each subunits of DNA-PK (each 200 µg/ml) for 30 min at 37°C. The AAV DNA replication assay was performed as described previously [25]. Briefly, preincubated Ad-infected HeLa cell extract with antibodies was incubated in a reaction buffer containing 30 mM HEPES (pH 7.5), 7 mM MgCl_2_, 0.5 mM DTT, 100 µM each dNTP, 25 µCi of [a-^32^P] dATP, 4 mM ATP, 40 mM creatine phosphate, 1 µg of creatine phosphokinase, 0.1 µg of NE substrate DNA, and 1 to 80 U of Rep68 baculovirus extract for 4 h at 37°C. After incubation, the reaction mixture was treated with Proteinase K and extracted with phenol/chloroform. DNA was precipitated in ethanol. The DNA was digested with *Dpn*I to remove bacterial DNA and was separated by 0.8% agarose gel. X-ray film was exposed on a dried gel.

### Immunodepletions

In order to deplete DNA-PK subunits, 25 mg of Ad-infected HeLa cell extract was mixed with 30 µl of anti-DNA-PKcs (DNA- PKcs Ab-4_cocktail_), anti-Ku70/80 (Ku Ab-3) or control antibody (mouse IgG2a Ab-1, Neomarkers) and incubated at 4°C overnight. Each reaction was added with 50 µl of ProteinG-agarose (Calbiochem) and incubated at 4°C overnight. DNA-PK subunits interacted with the agarose beads were pelleted by centrifugation. Supernatants were harvested carefully. This procedure was repeated three times. The supernatant was used for in vitro AAV replication studies as described above after confirm the DNA-PK depletion by Western blot analysis.

### Construction of synthetic AAV-ITR

A T-shaped AAV ITR was built by annealing and ligating three synthetic oligos, ITR 1 (65-mer): 5′-pGCTCGCTCACTGAGGCCGCCCGGGCAAAGCCCGGGCGTCGGGCGACCTTTGGTCGCCCGGCCTCA-3′, ITR 2 (35-mer): 5′-pGTGAGCGAGCGAGCGCGCAGAGAGGGAGTGGCCAA-3′, ITR 3 (19-mer): 5′-pTTGGCCACTCCCTCTCTGCGCGCTC-3′ (Figure 6A). This ITR was ligated with biotinated oligonucleotides, which can bind to streptavidin coated magnetic particles (Roche Inc).

### Assay for interaction between DNA-PK and AAV ITR DNA

AAV ITR on magnetic bead was incubated with purified DNA-PK (containing DNA-PKcs, Ku80 and Ku70), HeLa nuclear extract or Rep78 at room temperature or 37°C. The incubation was performed in one of two buffers overnight containing either 25 mM HEPES (pH7.5), 5 mM MgCL2, 1 mM DTT and 1% BSA or 20 mM HEPES (pH7.6), 1 mM EDTA, 10mM NH4SO4, 1mM DTT, 0.2% Tween 20 and 30 mM KCL. After incubation, the magnetic beads were held by a magnet and washed 3 times with the binding buffer. AAV-ITR binding proteins were eluted out, dialyzed overnight following the instruction of the kit, and subjected to western blot analysis.
